# Dynamin-2 Function and Dysfunction Along the Secretory Pathway

**DOI:** 10.3389/fendo.2013.00126

**Published:** 2013-09-18

**Authors:** Arlek M. González-Jamett, Fanny Momboisse, Valentina Haro-Acuña, Jorge A. Bevilacqua, Pablo Caviedes, Ana María Cárdenas

**Affiliations:** ^1^Facultad de Ciencias, Centro Interdisciplinario de Neurociencia de Valparaíso, Universidad de Valparaíso, Valparaíso, Chile; ^2^Programa de Anatomía y Biología del Desarrollo, ICBM, Facultad de Medicina, Departamento de Neurología y Neurocirugía, Hospital Clínico Universidad de Chile, Universidad de Chile, Santiago, Chile; ^3^Programa de Farmacología Molecular y Clínica, ICBM, Facultad de Medicina, Universidad de Chile, Santiago, Chile

**Keywords:** dynamin-2, endocytosis, exocytosis, actin, microtubules, mutations, Charcot–Marie–Tooth neuropathy, centronuclear myopathy

## Abstract

Dynamin-2 is a ubiquitously expressed mechano-GTPase involved in different stages of the secretory pathway. Its most well-known function relates to the scission of nascent vesicles from the plasma membrane during endocytosis; however, it also participates in the formation of new vesicles from the Golgi network, vesicle trafficking, fusion processes and in the regulation of microtubule, and actin cytoskeleton dynamics. Over the last 8 years, more than 20 mutations in the dynamin-2 gene have been associated to two hereditary neuromuscular disorders: Charcot–Marie–Tooth neuropathy and centronuclear myopathy. Most of these mutations are grouped in the pleckstrin homology domain; however, there are no common mutations associated with both disorders, suggesting that they differently impact on dynamin-2 function in diverse tissues. In this review, we discuss the impact of these disease-related mutations on dynamin-2 function during vesicle trafficking and endocytotic processes.

## Introduction

Dynamin was identified for the first time almost 25 years ago as a 100 kDa microtubule-associated protein that induced microtubule bundles and promoted microtubule sliding *in vivo* (1). As described by the same authors, the motor activity of dynamin required ATP and other co-purified polypeptides (1). A year later, the same group cloned and sequenced dynamin, and found that it contained a consensus GTP-binding site (2), and subsequently characterized its GTPase activity (3). At present, three dynamin isoforms encoded by three distinct genes (*DNM1*, *DNM2*, and *DNM3*) have been described in mammals (4). These exhibit approximately 80% homology in their sequences, yet they differ in their tissue expression pattern; dynamin-1 is mainly expressed in neuronal tissue, dynamin-2 is ubiquitously expressed, and dynamin-3 is expressed in brain, testis, and lungs (5). Of these three dynamin isoforms, only dynamin-2 appears to play a pleiotropic role during embryonic development (6). In fact, studies in knock-out animals show that deletion of dynamin-1 or -3 can be compensated by the other dynamin isoforms (7), while the deletion of dynamin-2 causes early embryonic lethality (8). Moreover, as discussed below, mutations in *DMN2* result in severe hereditary neuropathies and myopathies in humans, strongly suggesting that dynamin-2 has more susceptible functions in the nervous and skeletal muscle tissues.

**Figure 1:**
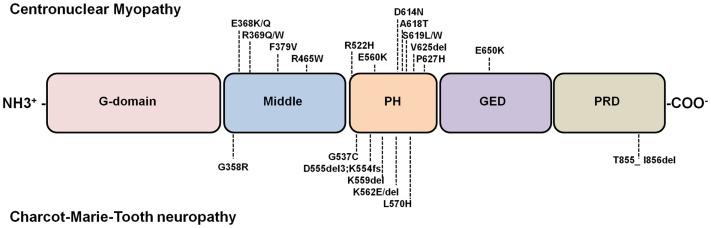
**Diagram of dynamin structure and localization of dynamin-2 mutations linked to CNM and CMT**. Dynamins are multimodular proteins comprising five highly conserved structural domains: a large N-terminal GTPase domain (G-domain), a middle domain, a PH domain that bind phosphoinositides, a GTPase effector domain (GED), and a C-terminal proline rich domain (PRD) that interacts with SH3-domain containing proteins. Most common disease-related dynamin-2 mutations are represented. Note that almost all dynamin-2 mutations identified in CNM and CMT patients are clustered into the PH domains; only one CNM-linked mutation has been found in GED, and one related to CMT has been identified in the PRD. The D555del3 mutation is one of the products of a 9-bp deletion in the exon 14 (1652_1659 + 1delATGAGGAGg) of the dynamin-2 gene (9). This gene deletion also results in a 65-kDa truncated protein (9). For that reason here is described as D555del3, K554fs. An updated database of DNM2 mutations is at the website www.umd.be/DNM2/ (10).

All dynamin isoforms exhibit at least four alternatively spliced variants, resulting in different dynamin proteins (5) that share a primary structure comprising: a large amino-terminal GTPase domain (G-domain) that binds and hydrolyzes GTP; a middle and a GTPase effector domains (GED) that form a “stalk” structurally essential region; a pleckstrin homology domain (PH) that binds inositol phospholipids and a carboxy-terminal proline and arginine rich-domain (PRD) that allows interaction with SH3-domain-containing-proteins (5) (Figure 1).

Dynamin function relies on its ability to form high order oligomers, and its self-assembly is necessary to promote its catalytic activity. Purified dynamin has been shown to spontaneously polymerize in the presence of negatively charged tubular templates such as lipid membranes (11), microtubules (3, 12), or actin bundles (13, 14) as well as after incubation in low ionic strength solutions (15). Over the last years several cryo-electron microscopy (16–18) and X-ray crystallographic studies (19, 20) of dynamin and its domains (21–24) have allowed a better understanding of the mechanisms mediating dynamin oligomerization. It appears that the stable dimers formed by the crossed interaction between the “stalk” regions of monomeric dynamins (20, 25) are the basic unit that allows dynamin polymerization (18), thus promoting the GTPase activation required for membrane remodeling and scission in different cellular processes.

In the present review, we discuss the different roles of dynamin during endocytosis, vesicle trafficking, and exocytosis, specially focusing in dynamin-2, and how disease-linked mutations in dynamin-2 gene alter such cellular processes.

## Dynamin as a Key Component of Endocytosis and Vesicle Recycling

Dynamin is a GTPase that plays a crucial role in the recycling of secretory vesicle in neuroendocrine cells (26).

The first evidence suggesting a role for dynamin in endocytosis came from the mapping and characterization of the *Drosophila shibire* gene, which was identified to be the *Drosophila* homolog of mammal dynamin (27, 28). *Drosophila* bearing mutations in the *shibire* gene exhibited a rapid and reversible paralysis at temperatures exceeding 29°C (29). The first ultrastructural analyses of synaptic terminals of *shibire* mutants showed a decreased number of synaptic vesicles and accumulation of “collared pits” suggesting a blocked step in the endocytotic process (30, 31). These ultrastructural changes were also observed in garland cells, a type of cell considered to be very active in endocytosis, where horseradish peroxidase uptake activity was also reduced, thus confirming an alteration in endocytosis in the *shibire* mutants (32). The role of dynamin in endocytosis in mammalian cells was later demonstrated using dynamin mutants with reduced GTPase activity (33, 34). The use of the non-hydrolyzable GTP analog GTP-γ-S allowed the visualization of endocytotic pits with elongated necks, decorated by electron-dense rings positive for dynamin immunoreactivity, showing that dynamin oligomerizes around the neck of endocytotic pits (35). Moreover, the fact that the dynamin mutant K44A, defective in GTP binding and hydrolysis, specifically blocked the coated vesicle formation without affecting the coat assembly and invagination revealed that dynamin is required for the constriction and subsequent budding of the coated vesicles (36). However, later studies suggested that dynamin also plays a role during a pre-collar stage, when the clathrin lattice is growing (37, 38). In this stage, dynamin may function as a scaffolding molecule that interacts with SH3 domain-containing proteins that control coated pit assembly and maturation (37). The recruitment of dynamin at endocytosis sites (39) also depends on its interaction with SH3-domain-containing proteins and phosphoinositides present at the plasma membrane via its PRD (40) and PH domain, respectively (41).

Regarding the mechanism by which dynamin catalyzes membrane fission, it has been proposed that the assembly of dynamin into helical oligomers around the neck of clathrin-coated pit promotes the dimerization of G domains of adjacent helical rungs, leading to the hydrolysis of GTP (18). The GTP hydrolysis triggers a conformational change in the dynamin polymer, allowing the constriction of the dynamin ring (18, 42, 43). The ring constriction strength then drives the constriction of the membrane neck, increasing membrane curvature (44). Such change in membrane curvature raises the local elastic energy, reducing the energy barrier to fission, and subsequently triggering the spontaneous fission at the boundary between the dynamin ring and the bare membranes (44).

The ability of dynamin to catalyze membrane fission is not only required in CME, but is also needed in other types of endocytotic pathways that are independent of clathrin. For instance, dynamin is required for caveolae-mediated endocytosis (45), which is essential for the uptake of molecules such as the complement C5b-9 complex (46). Dynamin also participates in the internalization of the β-chain of the interleukin-2 receptor through a clathrin independent, but RhoA dependent process, which is inhibited by the overexpression of a dominant negative mutant of dynamin GTPase activity (47). The entry in host cells of many pathogens and toxins such as anthrax toxin (48), Ebola virus (49), HIV (50), or Hepatitis C virus (51) also require the participation of dynamin.

Finally, the role of dynamin in vesicle formation is not only restricted to the plasma membrane and, as discussed below; its function is also needed in intracellular compartments.

## Role of Dynamin in Vesicle Trafficking and Golgi Function

Dynamin-mediated vesicle budding and membrane fission has also been reported in intracellular compartments such as endosomes (52, 53) and Golgi complex (54). Regarding dynamin-2 participation in vesicle trafficking from endosomes, this protein appears to play a role in two different steps: (1) the vesicle transport from late endosomes to the Golgi compartment (52) and (2) the recycling pathways from early endosomes (53). However, the establishment of dynamin-2 participation in the post-Golgi vesicle trafficking has been more controversial. Pioneer reports showed that the transport of vesicles from the Golgi to the cell surface or to lysosomes was independent of dynamin (36), and according to these results, no evidence of endogenous dynamin-2 localization in the Trans-Golgi network (TGN) nor of its participation in vesicle formation from this compartment were observed using different cell lines (55). Nevertheless, contemporary studies showed that ectopically expressed dynamin-2 localizes in the TGN in hepatocytes (54) and that the formation of clathrin-coated pits from Golgi membranes, in a cell-free assay, was inhibited in the presence of anti-dynamin antibodies, thus indicating the importance of dynamin at this level (54). Furthermore, canine kidney cells expressing a GTPase defective dynamin-2 mutant showed a restricted traffic of the protein p75 from the Golgi to the apical membrane (56). Also, the overexpression of a dominant negative mutant of dynamin-2 was shown to lead to the retention of proteins and accumulation of cisternae at the Golgi compartment, suggesting a role of dynamin-2 in keeping both the structure and function of the TGN (57).

In agreement with a role of dynamin-2 in Golgi vesicle formation, it was demonstrated that endogenous dynamin-2 localizes in the TGN in neuroendocrine mouse pituitary corticotrope cells (58), where it interacts with the βγ subunit of G-proteins via its PH domain. Interestingly, the overexpression of the purified PH domain induced the translocation of dynamin-2 from the Golgi complex to the plasma membrane, increasing receptor-mediated endocytosis but inhibiting basal and CRH-induced secretion of β-endorphins, suggesting a key role of dynamin-2 in the secretory pathway (58). Several studies have highlighted the importance of dynamin-2 to the proper traffic of nascent proteins from the TGN to the plasma membrane, a process that seems to be dependent on the actin cytoskeleton. In this regard, a subset of actin filaments anchored to the Golgi membrane via the small GTPase Arf-1 appears to form complexes with dynamin-2 and the actin-binding-protein cortactin, allowing the emergence and post-Golgi trafficking of secretory vesicles (59). Other proteins such as LimK1 and its substrate cofilin (60), syndapin, and the Wiskott–Aldrich-Syndrome-protein (WASP) (61) have been also involved in the regulation of the peri-Golgi actin cytoskeleton and in the dynamin-mediated transport of secretory vesicles from Golgi to the plasma membrane. Additionally, dynamin-2 function is necessary for the Golgi fragmentation and vesicle segregation induced by cholesterol in HeLa cells (62) and for the Golgi vesiculation induced by the c-SRC kinase activation (63) further supporting a pivotal role of dynamin in the Golgi dynamics along the secretory pathway.

## Dynamin as a Facilitator of Membrane Fusion

Dynamins have been involved in different types of fusion processes. Among them regulated exocytosis in neuroendocrine cells (64–69), acrosomal reaction (70), cell-to-cell fusion (71, 72), and fusion of virus with host cells (73, 74).

The first evidences showing the involvement of dynamin in exocytosis came from experiments performed at the beginning of 2000, which suggested that dynamin was involved in kiss-and-run, a transient mode of exocytosis, in neuroendocrine cells (64–66). In this type of exocytosis, the vesicle partially releases its content, and then it is recovered intact (75, 76). It was then hypothesized that dynamin would allow the reclosure of the vesicle mouth, acting through a mechanism similar to that described for vesicle formation during endocytosis (77). However, the mechanism by which dynamin controls the quantal release of hormones still remains unclear. More intriguing is the mechanism by which dynamin facilitates the fusion process during exocytosis. For instance, dynamin-2 reportedly favors granule secretion in both natural killer (78) and insulin-secreting cells (79). More recently, it was reported that, in chromaffin cells, dynamin-1 speeds up the expansion of the fusion pore, an intermediate structure formed during exocytosis, in a GTPase activity-dependent fashion (68). Therefore, both dynamin-1 and -2 appear to be involved in fusion processes. A possible explanation for this function is that dynamin interacts with SNARE proteins or SNARE-interacting proteins. In this regard, dynamin-2 has been shown to associate to secretory granules in chromaffin cells (80) via its interaction with syntaxin (80) and synaptophysin (67). In mammalian sperm, dynamin-2 associates with the SNARE regulatory protein complexin I (81), where it favors membrane fusion events during acrosomal exocytosis (70). In yeast, the dynamin homolog Vps1p interacts with the t-SNARE Vamp3; the disruption of this association with an antibody against Vps1p inhibits the fusion reaction (82). More recently, Peters’ lab has demonstrated that Vps1 binds to the Qa SNARE Vamp3 and controls trans-SNARE formation, which is essential for membrane fusion in yeasts (83).

An alternative explanation is that dynamin controls fusion events via actin cytoskeleton dynamics. In this regard, cortical actin is dynamically rearranged during regulated exocytosis in neuroendocrine cells acting as a barrier as well as a carrier for the access of the secretory granules to the plasma membrane (84–86). Actin filaments also control the fusion pore expansion (87), as dynamin does (68). As we discuss below, given that dynamin regulates actin organization (13, 14, 88), it is plausible to assume that its actions on fusion processes relay on its ability to modulate actin dynamics. According to this idea, we have recently demonstrated that endogenous dynamin-2 directs a Ca^2+^-dependent polymerization of cortical actin in adrenal chromaffin cells. Interestingly, both cortical actin and dynamin-2 regulate the initial fusion pore expansion and quantal release of transmitters, suggesting a synergistic action during exocytosis (89). Corey Smith and Collaborators found that the fusion pore expansion in chromaffin cells is controlled by the association of dynamin-1 with syndapin (69), a protein that modulates actin polymerization through neural-Wiskott–Aldrich-syndrome-protein (N-WASP) (90).

The role of dynamin-2 in fusion processes has also been extended to the fusion of myoblasts to form multinucleated myotubes (71). Interestingly, the GTPase activity of dynamin-2 is required at a stage that follows hemifusion but precedes the expansion of the fusion pores (71). The underlying mechanism is still unclear, but it could explain the muscular dysfunction in centronuclear myopathies caused by dynamin-2 mutations. Dynamin-2 has also been involved in the cell-to-cell fusion triggered by HIV-1 virus infection (72) and in fusion between HIV-1 virus and endosomes (73, 74). The latter authors proposed that dynamin promotes the expansion of the fusion pore that connects the HIV-1 envelope and the endosomal membrane, but the possible mechanism remains to be clarified.

Taken together, these findings indicate a pleiotropic role of dynamin in membrane fusion. Although dynamin does not promote membrane fusion by itself, it seems to act after a hemifusion state, facilitating the expansion of the fusion pore (71). The underlying mechanism probably relies on dynamin ability to sense membrane curvature and remodel membranes (91).

## Dynamin as a Regulator of Microtubule Instability

Although dynamin was first identified as a microtubules-associated protein (1) its specific role in microtubule integrity and dynamics is still unclear. Using papain-digestion experiments *in vitro*, Herskovits and Co-workers observed that the entire PRD constituted a microtubule-binding site (34). Later, it was demonstrated that only the PRD amino-terminal region was necessary for the association of dynamin to microtubules, whereas its C-terminus appeared to negatively regulate this interaction (92). Pioneering studies also described that dynamin polymerized around microtubules, interconnecting them and allowing bundle formation (1), and in turn, microtubules seemed to stimulate dynamin GTPase activity (12). Other findings showed that the middle domain of dynamin-2 binds to γ-tubulin (93) locating dynamin-2 to the centrosome, therefore suggesting its participation during centrosome cohesion (93). More recently, dynamin-2 was found to be enriched in microtubule bundles at the mitotic spindle of mitotic cells, playing a key role during the cell cycle progression (94). In 2009, Tanabe and Takei observed that depletion of dynamin-2 in COS-7 cells, led to an abnormal accumulation of stable microtubules, consequently inducing disturbance of the microtubules-dependent membrane trafficking. These authors did not find alterations in microtubule assembly in cells that expressed a specific interfering RNA against dynamin-2, but they did report increased stability of pre-existing microtubules, suggesting a role of dynamin-2 in microtubule dynamics instability (95). This dynamin role appears to be independent of its GTPase activity. On the other hand, a deletion of three amino acid residues located at the β3/β4 loops of the PH domain induced decoration of microtubules and accumulation of acetylated tubulin, indicating that this region is required for the correct bundling of microtubules (95). The authors also proposed that this action of dynamin-2 on microtubule instability is necessary for a proper formation of Golgi network and vesicle trafficking.

## Dynamin as a Director of Actin Cytoskeleton Dynamics

Dynamin-2 is recruited to different actin-rich structures such as phagocytic cups (96), podosomes (97), *Listeria* actin (98), lamellipodia (99), and filopodia (88), supporting a connection between dynamin-2 function and actin cytoskeleton polymerization. Moreover, both actin and dynamin-2 have regulatory functions during T cells activation (100), phagocytosis (101), and clathrin-dependent endocytosis (102). In the latter, actin and dynamin-2 have been shown to exhibit a synergistic action, where one modulates the recruitment of the other, and both participate in membrane remodeling and scission (103, 104).

In all these processes, dynamin appears to promote actin assembly in a manner dependent on its GTPase activity (14). The mechanism is still unclear, yet it is probably a consequence of its association with several nucleation promoting factors (NPF) via a PRD-SH3 interaction; among them Abp1 (105), the N-WASP (106), and cortactin (13, 88), all of which have been described as dynamin-partners during actin polymerization.

In 2010, Gu and Co-workers reported a direct interaction between dynamin and short actin filaments (F-actin). The latter promoted dynamin oligomerization, and in turn dynamin induced F-actin elongation (14). These authors suggested a model wherein dynamin would favor actin polymerization by removing the “caping”-protein gelsolin of the “barbed” ends (14). More recently, Yamada et al. (88) demonstrated that dynamin induces actin bundle stabilization in a way dependent on its association with cortactin in growth cone filopodia, where they seem to form ring-structures along actin filaments that stabilize F-actin bundles (88). Since dynamin oligomerization enhances its catalytic activity (14), its association with cortactin during actin polymerization fits better with its role as a GTPase than that of a mere “uncapping” protein.

## Impact of Disease-Related Dynamin-2 Mutations in Vesicle Trafficking and Endocytosis

Specific missense mutations and short deletions into the structural domains of dynamin-2 have been associated with two congenital autosomic neuromuscular disorders: Charcot–Marie–Tooth neuropathy (CMT) (9) and centronuclear myopathy (CNM) (107). While CMT is a demyelinating disease affecting peripheral nerves (108), CNM is characterized by a progressive weakness and atrophy of distal muscles, usually involving facial and extraocular musculature (109–111). Although most dynamin-2 mutations linked to CMT and CNM are located in the middle and PH domains, there are no common mutations to both disorders (112) and the molecular mechanisms that lead to the neuropathy or myopathy remain obscure. Figure 1 shows the localization of the CMT and CNM dynamin-2 mutations described until now, and Table 1 summarizes their impacts on different cellular processes.

**Table 1:** **Cellular function alterations induced by CNM and CMT mutants**.

Cellular process impaired	CNM mutations	CMT mutations
Intracellular calcium concentration in KI mice myofibers (113)	R465W	
Protein export from Golgi to plasma membrane in dynamin-2 (114)	E368K, R465W	D555del3, L570H
Clathrin-mediated endocytosis in:		
COS-1 and/or COS-7 cells (95, 112, 115)	R465W, R522H, E560K, S619L/W, V625del, P627H	K558E, K562E
RT4 Schwann cells and NSC-34 motor neurons (116)		G358R, G537C, K562E, K559del, L570H
Embryonic fibroblasts from homozygous KI mice (117)	R465W	
Clathrin-independent endocytosis (114)	E368K, R465W	D555del3, L570H
Raft-dependent endocytosis (114)	E368K, R465W	D555del3, L570H
Myelination in dorsal root ganglia (116)		G358R, G537C, K559del K562E, K562del, L570H
Demyelination in dorsal root ganglia (116)		G358R, K562E
Autophagy and autophagosome maturation (117)	R465W	

Regarding how these disease-related mutations affect dynamin-2 oligomerization and catalytic properties, *in vitro* studies have revealed that the CNM-linked mutations E368K/Q, R369Q/W, and R465W located into the middle domain of dynamin-2 (107) as well as the mutations A618T, S619L/W, and V625del clustered into the PH domain (118) exhibit an increased stability of the oligomers and an enhanced GTPase activity (119, 120). However, how these and other dynamin-2 mutations impact on dynamin-dependent cellular processes is still under discussion.

Given that the best-known role of dynamin-2 is to catalyze membrane scission during endocytosis, most of the studies regarding the mechanism underlying CNM associated to mutations in dynamin-2 have been focused in this cellular process. However, the findings are contradictory. The overexpression of the CNM-linked mutants R465W, R522H, S619L, P627H V625del, and E650K impaired CME in COS-1 and COS-7 cells (112, 115). The fact that the CNM mutant D614N causes intracellular mislocalization of both dynamin-2 and clathrin (121) suggests that an impairment in CME could be a consequence of the anomalous distribution of dynamin-2 and other endocytotic proteins. In opposition to the aforementioned findings, fibroblasts from patients harboring the mutations R465W or S619L reportedly display normal CME (112). In a similar manner, embryonic fibroblasts from heterozygous knock-in (KI) mice carrying the mutation R465W exhibited no defects in CME (113). Unlike the heterozygous R465W KI mice, the homozygous animals displayed impaired receptor-mediated endocytosis, indicating that this mutation differentially impacts on CME according to the heterozygous or homozygous carrier state. In this regard, fibroblasts from patients harboring the homozygous mutation F379V, the first one linked to a lethal congenital syndrome, also exhibited dysfunctional endocytosis (6). In order to compare the effects of dynamin-2 mutations associated to CNM in a context that mimics the homozygous and heterozygous states, Schmid and Collaborators (114) established stable cell lines from dynamin-2 conditional null mouse fibroblasts and expressed two CNM-linked dynamin-2 mutants (E368K and R465W). Cells expressing comparable levels of wild-type dynamin-2 and the given mutant were selected to evaluate clathrin-dependent and independent endocytosis, and also vesicle trafficking. The authors showed that none of these mutations affected CME in the condition that mimics the heterozygous state; and they were able to fully rescue transferrin uptake in dynamin-2 KO cells. Nevertheless, all these mutants impaired clathrin-independent endocytosis of epidermal grow factor receptors and raft-dependent endocytosis of cholera toxin. Furthermore, also in the condition that mimics the heterozygous state, all the mutants disrupted trafficking of p75/neurotrophin receptor from Golgi to plasma membrane. It seems therefore plausible that the physiological dysfunctions in CNM patients harboring heterozygous mutations in dynamin-2 are not a consequence of defective CME, but other dynamin-2-dependent processes, such as clathrin-independent endocytosis or vesicle trafficking could be affected in these conditions.

Regarding the mechanism underlying CMT associated to dynamin-2 mutations, Sidiropoulos and Co-workers (116) suggested that an impairment of CME in Schwanm cells plays an important role in the pathogenesis of CMT neuropathy. They showed that the CMT mutants K562E, G358R, G537C, and L570H, but not the CNM mutants R465W and E560K, strongly reduced myelination in dorsal root ganglia explant cultures derived from heterozygous embryos carrying a dynamin-2 null allele, thus mimicking the heterozygous state. These CMT mutants also reduced transferrin internalization in Schwann cells and motor neurons. Conversely, none of the 11 CNM mutations evaluated in that work had any effect on transferrin internalization, neither in Schwann cells nor in motor neurons, suggesting that CMT and CNM mutations affect different cellular processes.

The aforementioned cellular impairments induced by CNM- and CMT-mutations, as well as others not previously mentioned, are summarized in the Table 1.

## Concluding Remarks

The secretory pathway plays a pivotal role in mammalian cell function. Its disruption underlies many known diseases, such as those described herein. Dynamin-2 is critical in such mechanisms, as it regulates Golgi structure and function, cytoskeletal integrity, vesicle trafficking, and most interestingly, membrane fusion.

In the last 10 years, more than 20 disease-linked mutations have been reported in the dynamin-2 gene, affecting different dynamin-mediated cellular processes. Nevertheless, their impact on neuroendocrine tissues remains obscure. Also elusive are the mechanisms by which the said mutations affect actin cytoskeleton dynamics and exocytosis, processes which are dynamin-dependent and critical in the course of the secretory pathway. The latter opens an interesting research field that could prove useful to understand the mechanisms involved in the pathogenesis of dynamin-2 disease-related mutations.

Several diseases caused by genetic modifications remain as the final frontier in medicine, and although pivotal hallmarks such as the sequencing of the human genome have been attained, we still have much to learn about the cellular and molecular consequences of diverse gene mutations, specially, those that are common in many tissues. In this regard, dynamin-2 is a ubiquitous protein with diverse pleiotropic roles, whose mutations cause tissue-specific phenotypes. The existence of pertinent cell and animal models will be extremely useful for better understanding how dynamin-2 mutations impact on its properties and related functions, and consequently in the identification of potential therapeutic targets.

## Conflict of Interest Statement

The authors declare that the research was conducted in the absence of any commercial or financial relationships that could be construed as a potential conflict of interest.
